# In Vivo Response of γδ T Cells and Macrophages to Non-Bilayer Phospholipid Arrangements in a Lupus-like Mouse Model

**DOI:** 10.3390/ijms26178680

**Published:** 2025-09-05

**Authors:** Iván Galarce-Sosa, Albany Reséndiz-Mora, Rodrigo Ramos-Monteagudo, Giovanna Barrera-Aveleida, José Rundquist-Sánchez, Saúl Gómez-Manzo, Isabel Wong-Baeza, Carlos Wong-Baeza, Isabel Baeza

**Affiliations:** 1 Laboratorio de Biomembranas, Departamento de Bioquímica, Escuela Nacional de Ciencias Biológicas, Instituto Politécnico Nacional, Mexico City 11340, Mexico; ivan.gal27@hotmail.com (I.G.-S.); cbanyrm@icloud.com (A.R.-M.); rodrigoramosm1999@gmail.com (R.R.-M.); aveleida@gmail.com (G.B.-A.); jrundquist94@gmail.com (J.R.-S.); 2 Red de Salud del Instituto Politécnico Nacional, Mexico City 07320, Mexico; mwongb@ipn.mx; 3 Laboratorio de Bioquímica Genética, Instituto Nacional de Pediatría, Secretaría de Salud, Mexico City 04530, Mexico; saulmanzo@ciencias.unam.mx; 4 Laboratorio de Inmunología Molecular II, Departamento de Inmunología, Escuela Nacional de Ciencias Biológicas, Instituto Politécnico Nacional, Mexico City 11340, Mexico

**Keywords:** systemic lupus erythematosus, γδ T cells, macrophages, lipid antigens, non-bilayer phospholipid arrangements, anti-lipid antibodies

## Abstract

Anti-lipid autoantibodies are produced in systemic lupus erythematosus (SLE). These antibodies are associated with clinical manifestations of the disease, such as thrombosis, cardiovascular events, and neurological disorders. However, the cellular and molecular mechanisms that lead to the production of these antibodies are not well known. We developed a mouse model of lupus by administering liposomes bearing non-bilayer phospholipid arrangements (NPA) stabilized by chlorpromazine. These mice produce anti-NPA antibodies that trigger a lupus-like disease. In previous studies, we demonstrated that these antibodies are primarily produced by germinal centers and that NK1.1^+^ CD4^+^ T cells provide help to B cells, enabling them to produce these IgG antibodies. However, additional immune cells may contribute to the production of these antibodies. Therefore, in this work, we analyzed the in vivo responses of γδ T cells and macrophages in this mouse model. We found that γδ T cells from mice that produce anti-NPA antibodies produce IFNγ and IL-17, which can contribute to B cell class switching and production of anti-NPA IgG antibodies via germinal centers. Additionally, we found that macrophages are polarized into a proinflammatory M1 phenotype and produce IL-6 that can exacerbate inflammation and potentially lead to autoimmunity.

## 1. Introduction

Systemic lupus erythematosus (SLE) is a chronic, autoimmune, and inflammatory disease that affects multiple systems. It is characterized by the presence of autoantibodies and by the deposition of antigen–autoantibody complexes, which leads to tissue damage [1]. Anti-cardiolipin and anti-phosphatidylserine/prothrombin are the most common anti-lipid autoantibodies detected in SLE. These antibodies contribute to the inflammatory and thrombotic processes in SLE and are associated with clinical manifestations of the disease, such as thrombosis, cardiovascular events, and neurological disorders [1]. The clinical presentation of SLE is diverse, ranging from a mild skin disease (malar rash) to lupus nephritis or lupus-related kidney failure [2]. A study that compiled worldwide data for thirty years (1992–2022) found 5.14 new cases of SLE per 100,000 people per year, with a prevalence of 43.7 cases per 100,000 people [2]. Patients with SLE are treated with hydroxychloroquine and glucocorticoids due to their potent and rapid anti-inflammatory and immunosuppressive effects [3]. The etiology of SLE remains unclear, and the cellular and molecular mechanisms of the pathophysiology of this disease are not yet fully understood. However, it has been established that genetic predisposition, along with environmental, immunological, epigenetic, infectious, and hormonal factors, is involved in modulating SLE [4].

Lupus mouse models are classified as spontaneous, if the disease develops through genetic mutations, or induced, if the disease develops in response to a chemical compound [5]. NZB/NZW F1 and MRL/lpr mice are spontaneous lupus models; they produce autoantibodies, develop lymphoproliferative disorders, and exhibit skin and kidney pathologies [6]. The administration of pristane induces a lupus-like disease, with the production of autoantibodies and inflammation [7]. Our research group developed an induced lupus model by administering liposomes bearing drug-stabilized non-bilayer phospholipid arrangements (NPA) to mice [8,9]. NPAs are natural and transient lipid associations different from the lipid bilayer [10]. If NPAs are stabilized by drugs, such as chlorpromazine or procainamide, they become immunogenic and induce an immune response that leads to the production of anti-NPA antibodies [8,9]. These antibodies trigger a lupus-like disease in mice, because they bind to NPAs on cell membranes, leading to cell lysis and to the exposure of the cellular contents to the immune system, which in turn induces more autoantibodies [11]. IgM and IgG anti-NPA antibodies are present in the serum of patients with primary antiphospholipid syndrome and/or SLE, but not in the serum of healthy individuals [8,12].

Mice with the NPA-induced lupus model exhibit symmetrical facial lesions that resemble human malar erythema [9], and they have deposition of immune complexes in the basal membranes of their glomerular capillaries and mesangial matrix [8]. The first autoantibodies detected in these mice are anti-NPA antibodies, followed one month later by the detection of anti-cardiolipin, anti-histone, and anti-coagulant autoantibodies [8,9]. We have previously used this model to identify the immunological mechanisms that lead to the formation of anti-NPA antibodies, and found that anti-NPA IgG antibodies are mainly produced by germinal centers [13], and that NK1.1^+^ CD4^+^ T cells drive specific B cell activation and antibody class switching to IgG [14]. However, additional immune cells could participate in the induction of these antibodies. γδ T cells have a T cell receptor (TCR) that can bind to lipid antigens that are presented by CD1 [15,16]. Since γδ T cells can be activated by lipids, they are an interesting target in studying NPA immunogenicity. Activated γδ T cells produce interferon gamma (IFNγ), which leads to macrophage activation [17]. Macrophages respond to pathogens and secrete pro- and anti-inflammatory cytokines [18], and they also modulate the adaptive immune response by processing and presenting antigens [19] through the major histocompatibility complex (MHC) and CD1 molecules [20,21]. M1 macrophages produce proinflammatory cytokines and high levels of reactive nitrogen and oxygen species, and promote Th1 immune responses, while M2 macrophages regulate the immune response and promote tissue remodeling [18]. Therefore, in this study, we analyzed the role of γδ T cells and macrophages in the development of the lupus-like disease induced by stabilized NPA. We found that γδ T cells produce IFNγ and interleukin 17 (IL-17), which may promote B cell class switching and germinal center response to produce anti-NPA IgG antibodies. Additionally, macrophages are polarized into a proinflammatory M1 phenotype and produce interleukin 6 (IL-6), which enhances the immune response but can also cause tissue damage.

## 2. Results

### 2.1. Characterization of Smooth Liposomes and of Liposomes with Non-Bilayer Phospholipid Arrangements

**Figure 1:**
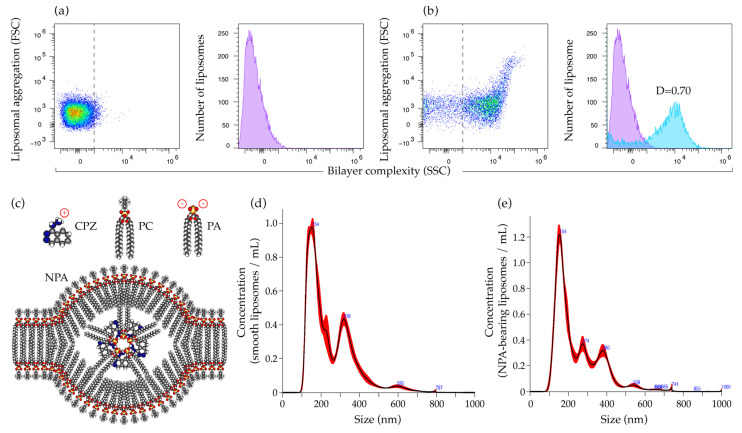
Characterization of smooth and NPA-bearing liposomes. Flow cytometry analysis of (**a**) smooth liposomes and (**b**) liposomes bearing NPA induced with 3 mM chlorpromazine. The purple histograms, as well as the dashed lines in the scatter plots, correspond to the complexity of the bilayer of the smooth liposomes. The blue histogram corresponds to the complexity of the bilayer of the NPA-bearing liposomes. The D value from the Kolmogorov–Smirnov test, which compares the complexity of the bilayer of both liposomes, is indicated. (**c**) Diagram of an NPA. Chlorpromazine (CPZ), an amphipathic drug with a triangular shape, interacts through its positive charge with phosphatidate (PA), a conical lipid with negative charges, to form an inverted micelle that is the center of the NPA. The lipids from the bilayer, such as phosphatidylcholine (PC), spread out over the inverted micelle and expose new epitopes to the immune system. Concentration versus size graphs for (**d**) smooth liposomes and (**e**) NPA-bearing liposomes were obtained with nanoparticle tracking analysis.

The formation of liposomes and the formation of NPA on liposomes were confirmed by flow cytometry. In accordance with previous studies [9], smooth liposomes constituted a homogeneous population with low complexity, which corresponds to the characteristic profile identified in previous studies (Figure 1a) [8,9]. We used 3 mM chlorpromazine to stabilize NPA on liposomes (Figure 1c). NPA-bearing liposomes showed an increase in bilayer complexity (D = 0.7) compared to smooth liposomes (Figure 1b). A value of D ≥ 0.5 indicates a statistically significant difference in bilayer complexity [22], which indicates the formation of NPA on liposomes.

To further characterize the liposomes, we used nanoparticle tracking analysis to determine the concentration and size distribution of smooth and NPA-bearing liposomes. Smooth liposomes had a concentration of 1.41 × 10^9^ liposomes/mL and a median size of 246.4 nm, while NPA-bearing liposomes had a concentration of 1.5 × 10^9^ liposomes/mL and a median size of 244.8 nm. The most abundant population of smooth liposomes and of NPA-bearing liposomes had a size of 154 nm (Figure 1d,e). The second most abundant population of smooth liposomes had a size of 318 nm (Figure 1d). This population was absent in NPA-bearing liposomes, where two populations with sizes of 274 and 380 nm were observed (Figure 1e). There were additional populations of both smooth and NPA-bearing liposomes, with very low concentrations (Figure 1d,e).

### 2.2. Mice Produce IgM and IgG Anti-NPA Antibodies in Response to Liposomes Bearing Non-Bilayer Phospholipid Arrangements

**Figure 2:**
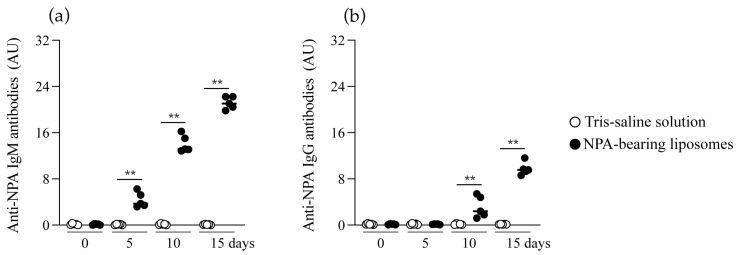
Mice that received NPA-bearing liposomes produce IgM and IgG anti-NPA antibodies. Detection of anti-NPA antibodies by liposomal ELISA. (**a**) IgM and (**b**) IgG anti-NPA antibodies were analyzed in the serum of mice before administration of the Tris–saline solution or NPA-bearing liposomes, and on days 5, 10, and 15 after administration. Antibody titers are shown as arbitrary units (AU). The Mann–Whitney test was used to determine significant differences between the study groups at the indicated times (** *p* ≤ 0.01).

Anti-NPA antibodies were analyzed in the serum of mice before and on days 5, 10, and 15 after Tris–saline solution or NPA-bearing liposomes administration. Before the administration of NPA-bearing liposomes or Tris–saline solution, no IgM or IgG anti-NPA antibodies were detected in mouse serum (Figure 2a,b). These antibodies were not detected throughout the study in mice that received Tris–saline solution (Figure 2a,b). In mice that received NPA-bearing liposomes, anti-NPA IgM antibodies were detected 5 days after liposome administration (Figure 2a), while anti-NPA IgG antibodies were detected by day 10 (Figure 2b). The IgM and IgG anti-NPA antibody titers increased over time (Figure 2a,b).

### 2.3. γδ T Cells Are Activated and Produce IFNγ and IL-17 in the Secondary Lymphoid Organs of Mice That Produce Anti-Non-Bilayer Phospholipid Arrangement Antibodies

**Figure 3:**
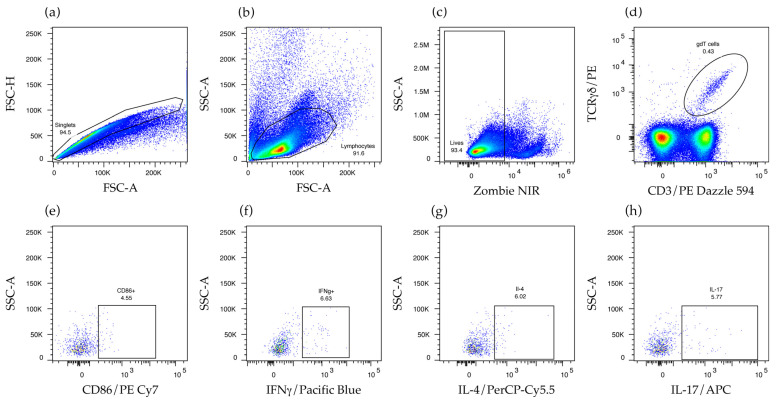
Flow cytometry strategy for the identification and characterization of γδ T cells. Cell suspensions were stained with specific antibodies against γδ T cells. First, (**a**) single events and (**b**) the lymphocyte gate were selected. Subsequently, (**c**) dead cells were excluded, and (**d**) γδ T cells positive for CD3 and TCR γδ, along with (**e**) activated γδ T cells positive for CD86, were selected. Finally, γδ T cells that produce (**f**) IFNγ, (**g**) IL-4, or (**h**) IL-17 were selected.

To assess the in vivo response of γδ T cells to NPA, we used flow cytometry to identify these cells in the spleen and the mesenteric lymph nodes of mice that received NPA-bearing liposomes or Tris–saline solution. To identify γδ T cells, single events were selected (Figure 3a), and then lymphocytes were selected according to their size and granularity (Figure 3b). Live cells, which exclude the Zombie NIR dye, were selected (Figure 3c), and subsequently, γδ T cells were identified by their expression of CD3 and TCR γδ (Figure 3d). Activated γδ T cells were also positive for CD86 (Figure 3e). Finally, the production of IFNγ, interleukin 4 (IL-4), or IL-17 (Figure 3f–h) by γδ T cells, and by other CD3^+^ γδ^-^ T, cells was also assessed.

**Figure 4:**
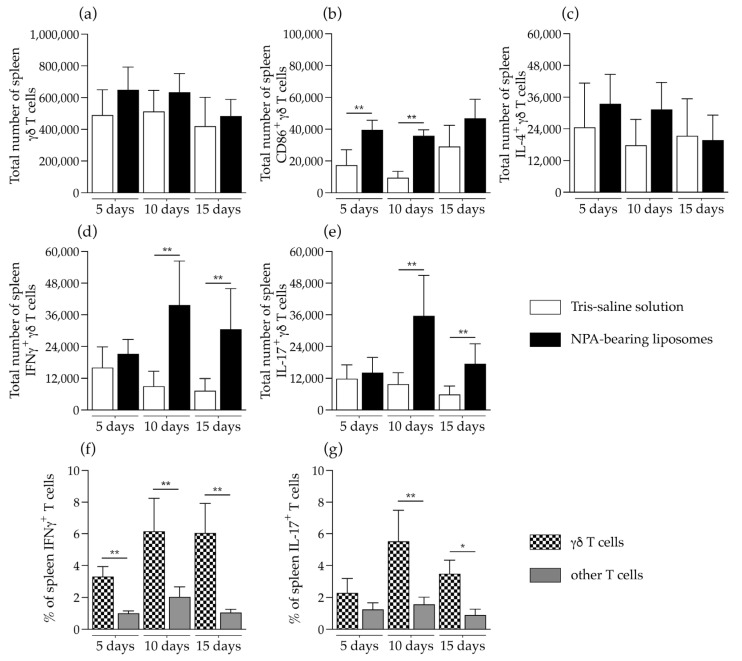
γδ T cells are activated and produce IFNγ and IL-17 in the spleen of mice that produce anti-NPA antibodies. Numbers of (**a**) total, (**b**) activated, and (**c**) IL-4-, (**d**) IFNγ-, or (**e**) IL-17-producing γδ T cells in the spleens of mice on days 5, 10, and 15 after the administration of Tris–saline solution or NPA-bearing liposomes. Percentage of γδ T cells and of other T cells that produce (**f**) IFNγ or (**g**) IL-17 in the spleens of mice on days 5, 10, and 15 after the administration of NPA-bearing liposomes. To determine statistically significant differences between study groups or between the percentage of γδ T cells and other T cells at a specific time, the Mann–Whitney test was used (* *p* ≤ 0.05; ** *p* ≤ 0.01). The spleens from five mice administered with NPA-bearing liposomes or with Tris–saline solution were analyzed for each condition at each time.

The total numbers of γδ T cells were not altered in the spleen of mice that received NPA-bearing liposomes, compared to mice that received Tris–saline solution (Figure 4a). However, these γδ T cells had increased levels of the activation marker CD86 on days 5 and 10 after liposome administration (Figure 4b). No significant differences in the number of IL-4-producing γδ T cells were detected (Figure 4c). On days 10 and 15 after the administration of NPA-bearing liposomes, a significant increase in the numbers of IFNγ- and IL-17-producing γδ T cells was detected, compared to mice that received Tris–saline solution (Figure 4d,e). In mice that received NPA-bearing liposomes, a statistically significant increase in the percentage of IFNγ-producing γδ T cells was detected on days 5, 10 and 15, compared to other IFNγ-producing T cells (Figure 4f). Additionally, on days 10 and 15, the percentage of IL-17-producing γδ T cells also showed a significant increase compared to other IL-17-producing T cells (Figure 4e).

**Figure 5:**
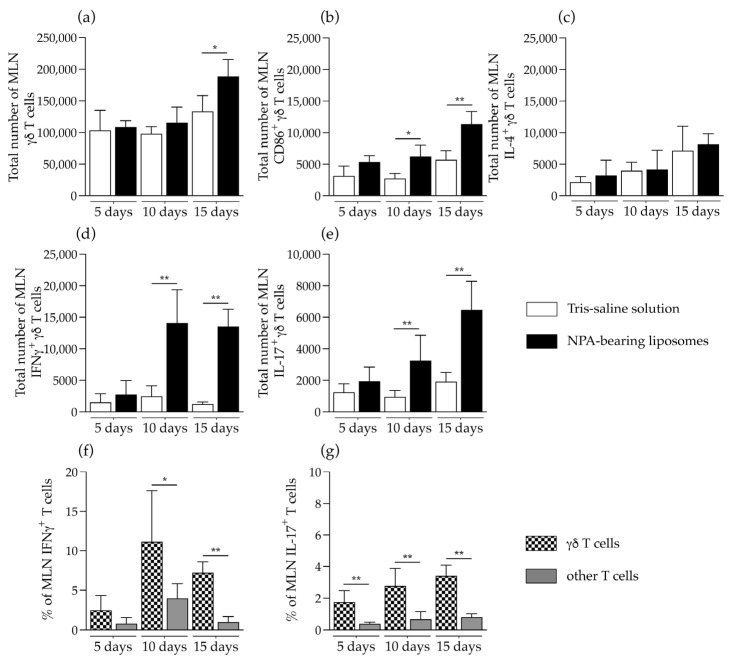
γδ T cells are activated and produce IFNγ and IL-17 in the mesenteric lymph nodes of mice that produce anti-NPA antibodies. Numbers of (**a**) total, (**b**) activated, and (**c**) IL-4-, (**d**) IFNγ, or (**e**) IL-17-producing γδ T cells in the mesenteric lymph nodes (MLN) of mice on days 5, 10, and 15 after the administration of Tris–saline solution or NPA-bearing liposomes. Percentage of γδ T cells and of other T cells that produce (**f**) IFNγ or (**g**) IL-17 in the MLN of mice on days 5, 10, and 15 after the administration of NPA-bearing liposomes. To determine statistically significant differences between study groups or between the percentage of γδ T cells and other T cells at a specific time, the Mann–Whitney test was used (* *p* ≤ 0.05; ** *p* ≤ 0.01). Five MLN from each of five mice administered with NPA-bearing liposomes or with Tris–saline solution were analyzed for each condition at each time.

γδ T cells were significantly increased in the mesenteric lymph nodes of mice that received NPA-bearing liposomes, on day 15 after liposome administration (Figure 5a). These γδ T cells also had increased levels of the activation marker CD86 on days 10 and 15 after liposome administration (Figure 5b). No differences were detected in the number of IL-4-producing γδ T cells (Figure 5c). A statistically significant increase in the number of IFNγ- and IL-17-producing γδ T cells was detected on days 10 and 15, compared to mice that received Tris–saline solution (Figure 5d,e). In mice that received NPA-bearing liposomes, a statistically significant increase in the percentage of IFNγ-producing γδ T cells was detected on days 10 and 15, compared to other IFNγ-producing T cells (Figure 5f). Additionally, on days 5, 10 and 15, the percentage of IL-17-producing γδ T cells also showed a significant increase compared to other IL-17-producing T cells (Figure 5e).

**Figure 6:**
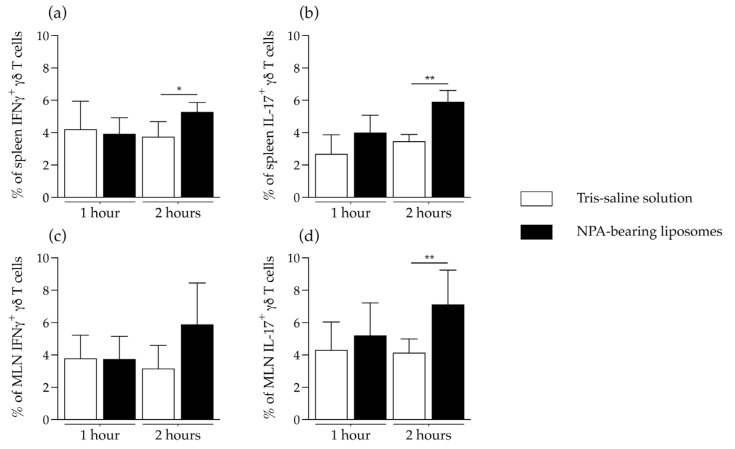
IFNγ and IL-17 production in response to NPAs by γδ T cells from mice that produce anti-NPA antibodies. Percentage of γδ T cells that produce (**a**,**c**) IFNγ or (**b**,**d**) IL-17 from the (**a**,**b**) spleens or the (**c**,**d**) mesenteric lymph nodes (MLN) from mice that received Tris–saline solution or NPA-bearing liposomes, after re-stimulation of the cell suspensions with NPA-bearing liposomes for 1 or 2 h. To determine statistically significant differences between study groups at a specific time, the Mann–Whitney test was used (* *p* ≤ 0.05; ** *p* ≤ 0.01). Five cell suspensions from spleen or MLN from each of five mice administered with NPA-bearing liposomes or with Tris–saline solution for 15 days were analyzed for each condition at each time.

To determine if γδ T cells can produce IFNγ or IL-17 in response to NPAs, we evaluated the in vitro response of γδ T cells from the spleen or the mesenteric lymph nodes of mice that produce anti-NPA antibodies, 15 days after the administration of NPA-bearing liposomes. After re-stimulation with NPA-bearing liposomes, the spleen cell suspensions from mice that received NPA-bearing liposomes had significant increases in the percentages of IFNγ- (Figure 6a) and IL-17-producing (Figure 6b) γδ T cells, compared to the spleen cell suspensions from mice that received Tris–saline solution. Additionally, mesenteric lymph node cell suspensions from mice that received NPA-bearing liposomes had a significant increase in the percentage of IL-17-producing γδ T cells (Figure 6d), but no significant difference in the percentage of IFNγ-producing γδ T cells (Figure 6c).

### 2.4. Liposomes Bearing Non-Bilayer Phospholipid Arrangements Induce M1 Macrophage Polarization in Mice Spleens

**Figure 7:**
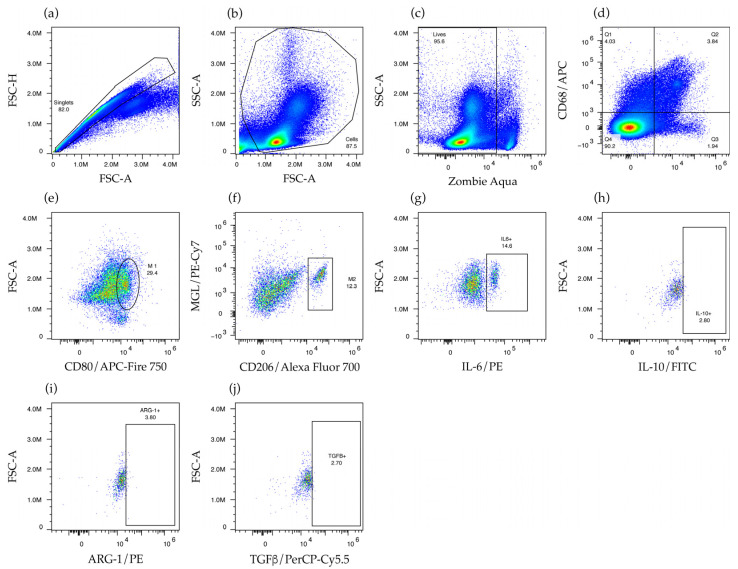
Flow cytometry strategy for the identification and characterization of macrophages. Cell suspensions were stained with specific antibodies against M1 and M2 macrophage markers. First, (**a**) single events were selected, then (**b**) cellular debris and (**c**) dead cells were excluded. Subsequently, (**d**) macrophages positive for CD68 and F4/80, along with (**e**) M1 macrophages positive for CD80 and (**f**) M2 macrophages positive for CD206 and macrophage galactose-type lectin (MGL), were selected. Finally, M1 macrophages that produce (**g**) IL-6 and M2 macrophages that produce (**h**) IL-10, (**i**) arginase 1 (ARG-1), or (**j**) TGFβ were selected.

To assess the in vivo response of M1 and M2 macrophages to NPA, we used flow cytometry to identify these cells in the spleen and the mesenteric lymph nodes of mice that received NPA-bearing liposomes or Tris–saline solution. To identify macrophages, single events were selected (Figure 7a), and then cellular debris was excluded (Figure 7b). Live cells, which exclude the Zombie Aqua dye, were selected (Figure 7c). Subsequently, macrophages were identified by their expression of F4/80 and CD68 (Figure 7d), along with M1 macrophages that express CD80 (Figure 7e) and M2 macrophages that express CD206 and macrophage galactose-type lectin (MGL) (Figure 7f). Finally, the production of IL-6 (Figure 7g) by M1 macrophages and the production of interleukin 10 (IL-10), arginase 1, or transforming growth factor beta (TGFβ) (Figure 7h–j) by M2 macrophages were also assessed.

**Figure 8:**
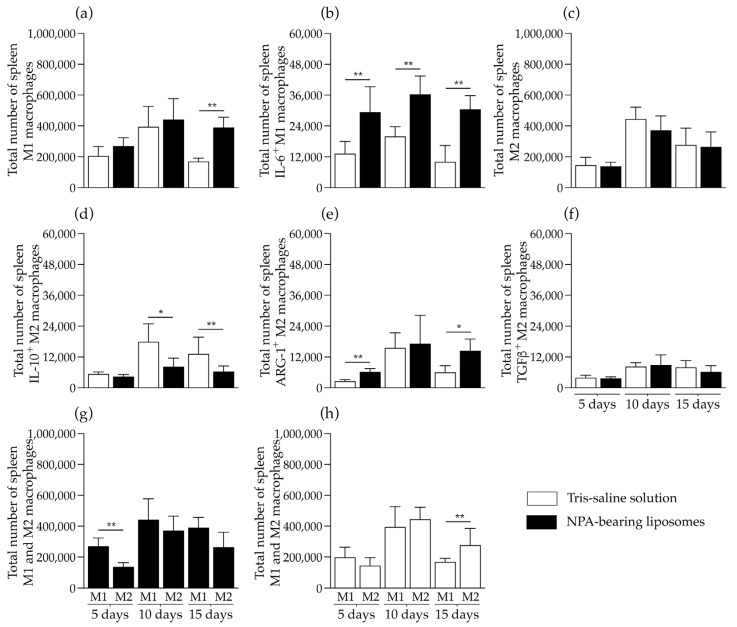
NPA-bearing liposomes induce M1 macrophage polarization in mouse spleens. Numbers of (**a**,**g**,**h**) M1 and (**c**,**g**,**h**) M2 macrophages, (**b**) IL-6-producing M1 macrophages, and (**d**) IL-10-, (**e**) arginase 1-(ARG-1), or (**f**) TGFβ-producing M2 macrophages from the spleens of mice on days 5, 10, and 15 after Tris–saline solution or NPA-bearing liposomes administration. To determine statistically significant differences between study groups at a specific time, or between M1 and M2 macrophages at a specific time within the same group, the Mann–Whitney test was used (* *p* ≤ 0.05; ** *p* ≤ 0.01). The spleens from five mice administered with NPA-bearing liposomes or with Tris–saline solution were analyzed for each condition at each time.

In the spleen of mice that received NPA-bearing liposomes, a statistically significant increase in the number of M1 macrophages was detected on day 15 (Figure 8a), as well as an increase in IL-6-producing M1 macrophages on days 5, 10, and 15, compared to mice that received Tris–saline solution (Figure 8b). In contrast, no statistically significant differences were detected in the number of M2 macrophages between the study groups (Figure 8c). However, a statistically significant increase in the number of IL-10-producing M2 macrophages was detected on days 10 and 15 in mice that received Tris–saline solution, compared to mice that received NPA-bearing liposomes (Figure 8d). A statistically significant increase in the number of arginase 1-producing M2 macrophages was detected on days 5 and 15 in mice that received NPA-bearing liposomes, compared to mice that received Tris–saline solution (Figure 8e). No differences were detected in the number of TGFβ-producing M2 macrophages (Figure 8f). A statistically significant increase in the number of M1 macrophages compared to M2 macrophages was detected on day 5 in the spleens of the mice that received NPA-bearing liposomes (Figure 8g). In contrast, an increase in M2 macrophages compared to M1 macrophages was detected on day 15 in the spleens of mice that received Tris–saline solution (Figure 8h).

**Figure 9:**
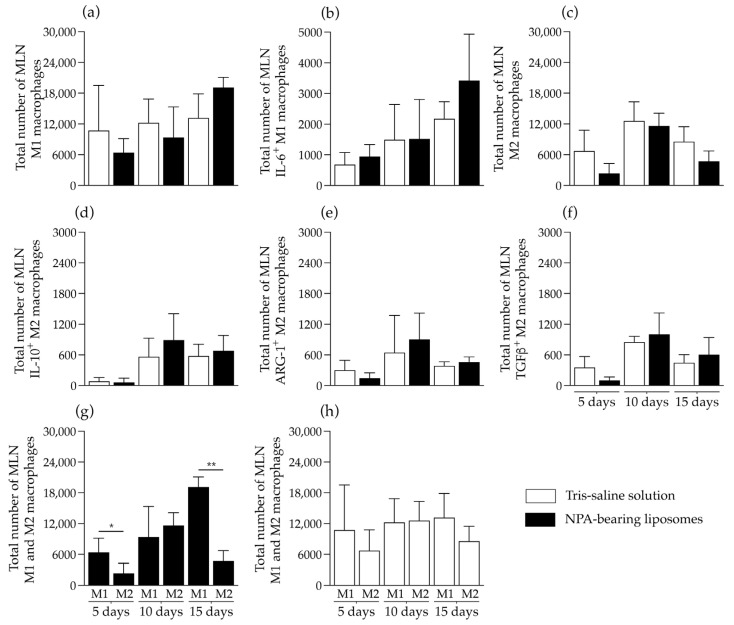
NPA-bearing liposomes do not induce macrophage polarization in the mesenteric lymph nodes of mice. Numbers of (**a**,**g**,**h**) M1 and (**c**,**g**,**h**) M2 macrophages, (**b**) IL-6-producing M1 macrophages, and (**d**) IL-10-, (**e**) arginase 1-(ARG-1), or (**f**) TGFβ-producing M2 macrophages from the mesenteric lymph nodes (MLN) of mice on days 5, 10, and 15 after Tris–saline solution or NPA-bearing liposomes administration. To determine statistically significant differences between study groups at a specific time, or between M1 and M2 macrophages at a specific time within the same group, the Mann–Whitney test was used (* *p* ≤ 0.05; ** *p* ≤ 0.01). Five MLN from each of five mice administered with NPA-bearing liposomes or with Tris–saline solution were analyzed for each condition at each time.

In the mesenteric lymph nodes of mice that received NPA-bearing liposomes, no differences were detected in the number of M1 macrophages (Figure 9a), M1 macrophages that produce IL-6 (Figure 9b), M2 macrophages (Figure 9c), and M2 macrophages that produce IL-10 (Figure 9d), arginase 1 (Figure 9e), or TGFβ (Figure 9f), compared to mice that received Tris–saline solution. However, in the mesenteric lymph nodes of mice that received NPA-bearing liposomes, a statistically significant increase in the number of M1 macrophages compared to M2 macrophages was detected on days 5 and 15 (Figure 9g), but no differences were detected in mice administered with Tris–saline solution (Figure 9h).

## 3. Discussion

The lupus mouse model induced by the administration of NPA-bearing liposomes has been previously characterized in the advanced stages of the disease. These mice develop a malar rash that sometimes resembles the butterfly wings of SLE patients, as well as glomerulonephritis and several autoantibodies [8,9]. Some of the molecular and cellular mechanisms that lead to the formation of anti-lipid IgG antibodies in this model have also been described [13,14]. In this study, the lupus mouse model was induced by the administration of liposomes bearing NPA stabilized with chlorpromazine, and its development was confirmed by detecting anti-NPA antibodies in mouse serum. To further understand how anti-NPA antibodies are generated and contribute to the pathogenesis of this lupus-like disease, we analyzed γδ T cells and macrophages at early stages (5, 10, and 15 days) of the model development. These innate immune cells, with their functional plasticity and diversity, respond very quickly to tissue disruptions. However, they can also participate in antigen presentation to the adaptive immune system [20,21,23,24]. Since γδ T cells can recognize lipid antigens and produce cytokines [16,25], we investigated their involvement in the generation of anti-NPA antibodies and in disease pathogenesis in this mouse model of lupus.

We detected an increased number of IFNγ- and IL-17-producing γδ T cells in the spleen and in the mesenteric lymph nodes of mice that produce anti-NPA antibodies, which is consistent with findings reported in patients with active SLE. These patients have increased blood γδ T cells [26,27], which produce IFNγ, IL-17, IL-4, and TGF-β [28]. In SLE patients, γδ T cells mainly participate in the chronic inflammation by secreting proinflammatory cytokines like IL-17, which promotes the recruitment of inflammatory cells that damage organs [29], and contributes to lupus nephritis [30]. The higher number of IFNγ-producing γδ T cells compared to IL-17-producing γδ T cells that we detected in these mice suggests a strong recognition of NPA lipids by γδ T cells, since it was reported that weak antigen recognition by γδ T cells leads to IL-17 production, while strong antigen recognition leads to IFNγ production [31,32]. In addition, the percentage of cytokine-producing γδ T cells was higher than the percentage of other cytokine-producing T cells (CD3^+^ γδ^-^ T), which are mainly αβ T cells, in the mice that received NPA-bearing liposomes. This suggests that, at the analyzed times, γδ T cells are the main T cells that respond to NPAs.

To determine if γδ T cells can directly respond to NPAs, we performed an in vitro re-stimulation assay. After re-stimulation with NPA-bearing liposomes, the spleen and mesenteric lymph node cell suspensions from mice that received NPA-bearing liposomes had significant increases in the percentages of cytokine-producing γδ T cells, compared to the cell suspensions from mice that received Tris–saline solution. These results suggest that mice that received NPA-bearing liposomes had primed NPA-specific γδ T cells, which produced IFNγ and IL-17 upon re-stimulation with NPAs.

IFNγ increases the expression of MHC class I and class II molecules, promotes Th1 responses and activates macrophages and cytotoxic T cells [17]. IFNγ levels are increased in mouse models of lupus, and these levels are associated with the development of autoantibodies and with disease progression [33]. IFNγ levels are also increased in patients with SLE [33]. IFNγ signaling on B cells through the IFNγ receptor (IFNγR) is required for the development of follicular T cells and germinal centers in lupus-prone B6.*Sle1b* mice [34]. B6.*Sle1b* mice with IFNγR-deficient B cells (B6.*Sle1b.*IFNγR^−/−^ mice) have smaller and fewer germinal centers than control B6.*Sle1b* mice, with reduced percentages of B220^+^PNA^hi^Fas^hi^ B cells and CD4^+^PD1^hi^CXCR5^hi^ follicular T cells. B6.*Sle1b.*IFNγR^−/−^ mice also had decreased serum levels of IgG2b and IgG2c anti-nucleosome, anti-histone and anti-dsDNA antibodies [34]. In another mouse model of B cell-driven autoimmunity, deletion of IFNγR on B cells also reduces the number of germinal centers and the serum levels of autoantibodies [35]. This study demonstrates that, in mice and human B cells, IFNγ signaling synergizes with B cell receptor, TLR7 or CD40 signaling to induce the expression of germinal center markers, such as BCL6, CD38 and Fas [35]. BCL6 is a transcription factor with a critical role in several stages of the germinal center reaction [36]. In our lupus mouse model, the production of anti-NPA IgG antibodies mainly occurs through germinal centers [13]. This suggests that the IFN-γ produced by γδ T cells in response to NPA could contribute to germinal center formation by acting directly on B cells (through IFNγR), leading to B cell activation, class switching, and production of anti-NPA IgG antibodies.

The IL-17 produced by γδ T cells in our lupus mouse model could cause inflammation, as is observed in humans and in mouse models of autoimmune diseases, where the early activation of γδ T cells generates IL-17 and this leads to an inflammatory response [37,38,39]. However, IL-17 also influences the adaptive immune response. IL-17 causes RelA upregulation in B cells, which enhances Rgs16 signaling and leads to B cell chemotaxis and germinal center formation [40]. Furthermore, IL-17 can act as a costimulatory signal to promote B cell proliferation and antibody class switching [41,42]. In mice immunized with myelin oligodendrocyte glycoprotein (MOG), Th17 cells increase the number of germinal centers and the serum levels of IgG1 and IgG2b anti-MOG antibodies [43]. Therefore, in our lupus mouse model, IL-17-producing γδ T cells could also activate B cells to induce the production of anti-NPA IgG antibodies via germinal centers. In contrast, we found no significant differences in the numbers of IL-4-producing γδ T cells in the spleen and in the mesenteric lymph nodes of these mice. However, in a previous study, we reported increased numbers of IL-4-producing NK1.1^+^, CD4^+^ T cells in the spleen and in the mesenteric lymph nodes of these mice [14]. IL-4 promotes antibody class switching [44], so this cytokine could also participate in the induction of anti-NPA IgG antibodies in this mouse model.

Our findings suggest that γδ T cells play a crucial role in inducing germinal centers through the production of IFNγ and IL-17. This is consistent with studies in γδ T cell-deficient (TCRδ^−/−^) mice, which have impaired germinal center formation, inefficient follicular T cell differentiation, and decreased serum levels of specific IgG antibodies after immunization [45,46]. In addition, the γδ T cells from our mice with the lupus-like disease could also directly interact with B cells in germinal centers, since γδ T cells can assume a function similar to that of follicular T cells, by expressing costimulatory molecules such as ICOS and CD40L, and by secreting cytokines such as IL-2, IL-4, IL-10, and IL-21 [47,48], which would promote the production of anti-NPA antibodies. However, to directly assess the role of γδ T cells in the induction of germinal centers that lead to the production of anti-NPA antibodies, future studies should be conducted in TCRδ^−/−^ mice.

Macrophages are phagocytic immune cells crucial for maintaining tissue homeostasis and for eliminating pathogens. They can develop proinflammatory (M1) or anti-inflammatory (M2) phenotypes [49,50]. We found that macrophages were polarized towards the proinflammatory M1 phenotype in the spleens of mice that produce anti-NPA antibodies. These M1 macrophages consistently produced IL-6 throughout the study. IL-6 enhances the immune response and can cause tissue damage if it is not adequately regulated. Furthermore, IL-6 is a crucial mediator in the differentiation of Th17 cells and in the suppression of regulatory T cells, which can exacerbate inflammation and potentially lead to autoimmunity [50,51,52]. IL-6 also promotes IL-17 production by γδ T cells [53,54]. The main cytokine that leads to M1 macrophage polarization is IFNγ [17], suggesting that, in our model, IFNγ-producing γδ T cells that respond to NPAs contribute to the polarization of macrophages towards the M1 phenotype, and then, the IL-6 produced by these macrophages contributes to IL-17 production by γδ T cells. Patients with SLE have increased levels of serum IL-6, compared to healthy controls [51]. An imbalance between the M1 and M2 phenotypes has also been associated with SLE progression [55]. In addition, myeloid cells from patients with inactive or active SLE have increased expression of genes associated with M1 macrophage polarization, compared to healthy controls [56]. Our results are in accordance with these findings.

In contrast to mice that received NPA-bearing liposomes, mice that received Tris–saline solution had increased M2 macrophages and IL-10-producing M2 macrophages in their spleens, which suggests an anti-inflammatory and immunoregulatory state that promotes immune tolerance and maintains tissue homeostasis. M2 macrophages are associated with tissue repair and with suppression of the inflammatory response, and IL-10 attenuates excessive immune activation, prevents tissue damage, and promotes the resolution of inflammation [49,57]. However, we detected an increase in arginase-1-producing M2 macrophages in mice that received NPA-bearing liposomes. These macrophages are associated with wound healing and immune regulation [58] but, in this mouse model, they are insufficient to induce an anti-inflammatory environment. We found no significant differences in TGFβ-producing M2 macrophages in the spleen or in the mesenteric lymph nodes of any of the analyzed mice.

Our results indicate that NPA-bearing liposomes induce M1 macrophage polarization in mice spleens, but not in mice mesenteric lymph nodes. We observed an increase in M1 macrophages compared to M2 macrophages 15 days after antigen administration, but there were no other differences in the numbers of M1 or M2 macrophages, or in the production of IL-6, IL-10, TGFβ or arginase-1, by the lymph node macrophages. The differential response of spleen and lymph node macrophages could be partially explained by the intraperitoneal administration of the NPA-bearing liposomes. These liposomes would reach the bloodstream mainly through the blood capillaries that perfuse the peritoneum, and to a lesser extent through the lymphatic capillaries [59]. From the bloodstream, the NPA-bearing liposomes would efficiently reach the spleen, leading to the activation of macrophages in this organ. However, other studies have reported functional differences between the macrophage subsets of the spleen and the macrophage subsets of the mesenteric lymph nodes [60], and this could also explain their differential responses to NPA-bearing liposomes. The spleen has marginal zone and red pulp macrophages. Since marginal zone macrophages recognize antigens and initiate innate immune responses [61], it is likely that these are the cells that respond to the NPA-bearing liposomes. Meanwhile, the mesenteric lymph nodes have sinusoidal subcapsular, medullary region and medullary cord macrophages [60,62], which are tolerogenic macrophage subsets that prevent excessive inflammation in the intestine, regulate homeostasis, and respond to antigens [62,63]. Our results indicate that these lymph node macrophage subsets have a limited response to NPA-bearing liposomes.

In summary, our findings suggest that γδ T cells play a crucial role in the early stages of the lupus-like disease by producing IFNγ and IL-17, which could contribute to the induction of germinal centers that produce IgG anti-NPA antibodies. In addition, we also detected IL-6-producing M1 macrophages in the early stages of this lupus-like disease. It is important to assess the role of these cells during the later stages of the lupus-like disease, to determine if they remain activated and contribute to chronic inflammation or antibody production, as is described in SLE patients [26,27,28,51,56].

## 4. Materials and Methods

### 4.1. Formation and Characterization of Liposomes Bearing Non-Bilayer Phospholipid Arrangements

Liposomes of egg yolk L-α-phosphatidylcholine (Sigma Aldrich, St. Louis, MO, USA) and egg yolk L-α-phosphatidate (Sigma Aldrich, St. Louis, MO, USA) (2:1 molar ratio) were formed by a modified reverse-phase evaporation method [8,14]. Phospholipids were dissolved in 1 mL of diethyl ether. Subsequently, 330 µL of Tris–saline solution was added, and the resulting mixture was sonicated three times in a G112SPI sonicator (Laboratory Supplies, Hicksville, NY, USA). Each sonication lasted 5 s, followed by a 30 s resting interval. Diethyl ether was completely removed under a stream of oxygen-free dry nitrogen at reduced pressure with an evaporator at 37 °C. Subsequently, Tris–saline solution was added to achieve a final volume of 1 mL. Finally, the smooth liposome suspension was filtered with a 0.45 µm Millipore membrane (Sigma Aldrich, St. Louis, MO, USA). To induce NPA on liposomes, 3 mM chlorpromazine (Sigma Aldrich, St. Louis, MO, USA) was added, and the liposomes were incubated for 30 min at 37 °C. Liposomes were analyzed in a FACSAria III flow cytometer (Becton Dickinson, San Jose, CA, USA), with size (FSC) and complexity (SSC) set in a logarithmic mode. A total of 30,000 events were acquired, and the data were analyzed with FlowJo software version 10.10 (Becton Dickinson, San Jose, CA, USA). Results are presented as histograms of liposomal complexity or as dot plots of liposomal size versus liposomal complexity. Liposome concentration per ml of suspension and liposome size were determined by nanoparticle tracking analysis in a Nanosight NS3000 (Malvern Panalytical, Malvern, UK), using NTA 3.2.16 software.

### 4.2. Immunization of Mice with Liposomes Bearing Non-Bilayer Phospholipid Arrangements

To analyze the in vivo response of γδ T cells and macrophages to NPA, we used two groups of 6-week-old female BALB/c mice. The first group received Tris–saline solution, and the second group received liposomes bearing NPA that were induced with 3 mM chlorpromazine. Before the administration of NPA-bearing liposomes or Tris–saline solution, mice received an intraperitoneal injection of 100 µL of complete Freund’s adjuvant (Sigma Aldrich, St. Louis, MO, USA) diluted 1:1 in Tris–saline solution. On day one, 50 µL of NPA-bearing liposomes or Tris–saline solution were administered intrasplenically, followed by intraperitoneal administrations of 100 µL of NPA-bearing liposomes or Tris–saline solution on days 4, 8, and 12. Five mice from each group were euthanized on days 5, 10, and 15 after the first liposome or Tris–saline solution administration. Spleens and mesenteric lymph nodes were extracted and placed in PBS (Gibco, Grand Island, NY, USA) with 1% bovine serum albumin (Biowest, Nuaillé, France) and 0.01% sodium azide (Sigma Aldrich, St. Louis, MO, USA). Finally, cell suspensions were obtained for subsequent flow cytometry analysis.

### 4.3. Detection of IgM and IgG Anti-Non-Bilayer Phospholipid Arrangements Antibodies

Mice were bled via the orbital sinus vein before antigen administration and on days 5, 10, and 15 after Tris–saline solution or liposome administration. The sera were aliquoted, incubated at 56 °C for 30 min to inactivate complement proteins, and stored at −20 °C. The liposomal ELISA technique, as described by our research group, was used to detect the presence of IgM and IgG anti-NPA antibodies [8,64]. 100 µL of a suspension containing NPA-bearing liposomes (0.1 μmol in 100 µL of TS) was added to each well of a 96-well flat-bottom plate (Costar Co., Cambridge, MA, USA). The plate was incubated for 12 h at room temperature. Subsequently, 200 µL of blocking solution (PBS with 4% fetal bovine serum) was added and incubated for 1 h at room temperature. After removing the blocking solution, 100 µL of mouse serum, diluted 1:50 with PBS, was added to each well and incubated for 1 h at 37 °C. The monoclonal antibody H308, specific to NPA, was used as a positive control, while sera from mice before liposome administration were used as negative controls. The blank well contained all the reagents, except for mouse serum. The plates were washed four times, followed by the addition of 100 µL of peroxidase-conjugated goat anti-mouse IgM or IgG antibody (Sigma Aldrich, St. Louis, MO, USA), diluted 1:2,000. The plates were incubated for 1 h at 37 °C. The plates were then washed four times, and 100 µL of a solution containing hydrogen peroxide (Sigma Aldrich, St. Louis, MO, USA) and the chromogen o-phenylenediamine (Sigma Aldrich, St. Louis, MO, USA) was added to each well. The mixture was incubated for 30 min at 37 °C, and the reaction was stopped by adding 2.5 M sulfuric acid. Absorbances at 492 nm were measured in a Multiskan ELISA reader (MTX Labsystems, Vienna, VI, USA). Results are presented in arbitrary units (AU), which were calculated as follows: AU = [(A_SAMPLE_ − A_BLANK_)/(A_POS_ − A_BLANK_)] (100). A_SAMPLE_ is the absorbance of the wells with mouse serum samples, A_BLANK_ is the absorbance of the blank well, and A_POS_ is the absorbance of the positive control well.

### 4.4. Analysis of γδ T Cells

Spleen and mesenteric lymph node cell suspensions were obtained from mice that received NPA-bearing liposomes or Tris–saline solution. The cell suspensions were filtered through a 70 µm cell strainer (Becton Dickinson, San Jose, CA, USA). Splenic red blood cells were removed using 2 mL of lysing solution (Becton Dickinson, San Jose, CA, USA) for 5 min at 4 °C, and the lysis was stopped with 5 mL of FACS solution. The viable cell count in the cell suspensions was determined using the trypan blue method in a Neubauer chamber. To evaluate cytokine production in γδ T cells, we added 0.7 µL/mL of monensin (Beckton Dickinson, San Jose, CA, USA) to the cell suspensions and incubated them for 4 h at 37 °C and 5% CO_2_. Subsequently, two million live cells were stained with anti-CD3/PE Dazzle 594 (clone: 17A2, isotype: rat IgG2b, κ) and anti-TCR γδ/PE (clone: GL3, isotype: hamster IgG2) (BioLegend, San Diego, CA, USA) to identify γδ T cells. Activated γδ T cells were evaluated using anti-CD86/PE Cy7 (clone: GL-1, isotype: rat IgG2a, κ) (BioLegend, San Diego, CA, USA). Anti-TCR γδ and anti-CD86 antibodies, along with their isotype controls, were used at a concentration of 5 mg/mL, whereas anti-CD3 antibody and its isotype control were used at 2.5 mg/mL. Cells were incubated with the antibodies at room temperature for 30 min in the dark, then fixed with 100 µL of Cytofix/Cytoperm (Beckton Dickinson, San Jose, CA, USA) at room temperature for 10 min. The cells were then washed and permeabilized with 100 µL of 1× PermWash (Beckton Dickinson, San Jose, CA, USA). Anti-IFN-γ/Pacific Blue (clone: XMG1.2, isotype: rat IgG1, κ), anti-IL-4/PerCP-Cy5.5 (clone: 11B11, isotype: rat IgG1, κ), and anti-IL17/APC (clone: TC11-18H10.1, isotype: rat IgG1, κ) antibodies (BioLegend, San Diego, CA, USA) diluted in PermWash at a concentration of 5 mg/mL were added. Cells were incubated with the antibodies at room temperature for 30 min in the dark and were then washed and fixed with 300 µL of 1% paraformaldehyde. Samples were kept at 4 °C in the dark until flow cytometry analysis, as described below.

### 4.5. In Vitro Re-Stimulation of γδ T Cells

Five BALB/c mice immunized as described above were euthanized 15 days after NPA-bearing liposomes or Tris–saline solution administration. Cell suspensions from the spleen and mesenteric lymph nodes were obtained, as described above. Then, one million cells were added to each well of a 24-well plate (Costar Co., Cambridge, MA, USA) containing RPMI 1640 supplemented with 5% complement-free fetal bovine serum (Gibco, Grand Island, NY, USA). Subsequently, NPA-bearing liposomes were added to a final concentration of 0.05 μmol, and the cells were incubated at 37 °C with 5% CO_2_ for 1 or 2 h. The IFNγ- and IL-17-producing γδ T cells were analyzed by flow cytometry, as described above.

### 4.6. Analysis of M1 and M2 Macrophages

Monensin-treated cell suspensions were obtained from the spleen and mesenteric lymph nodes of mice that received NPA-bearing liposomes or Tris–saline solution. Subsequently, two million live cells were stained with anti-F4/80/PE Dazzle 594 (clone: BM8, isotype: rat IgG2a, κ) and anti-CD68/APC (clone: FA-11, isotype: rat IgG2) (BioLegend, San Diego, CA, USA) to identify macrophages. M1 macrophages were evaluated with anti-CD80/APC/Fire 750 antibody (clone: 16-10A1, isotype: Armenian hamster IgG1) (BioLegend, San Diego, CA, USA), and M2 macrophages were evaluated with anti-CD206/Alexa Fluor 700 (clone: C068C2, isotype: rat IgG2a, κ) and anti-MGL/PE/Cyanine7 (clone: LOM-14, isotype: rat IgG2b, κ). Anti-F4/80 and anti-CD80 antibodies, along with their isotype controls, were used at a concentration of 5 mg/mL. Anti-CD68, CD206, and anti-MGL antibodies, along with their isotype controls, were used at 2.5 mg/mL. Cells were incubated with the antibodies at room temperature for 30 min in the dark, and were then fixed with 100 µL of Cytofix/Cytoperm (Beckton Dickinson, San Jose, CA, USA) at room temperature for 10 min. The cells were then washed and permeabilized with 100 µL of 1× PermWash (Beckton Dickinson, San Jose, CA, USA). Anti-IL-6/PE (clone: MP5-20F3, isotype: rat IgG1, κ), anti-arginase-1/PE (clone: W21047I, isotype: rat IgG2b, κ), anti-IL-10/FITC (clone: JES5-16E3, isotype: rat IgG2b, κ), and anti-TGF-β1/PerCP/Cyanine5.5 (clone: TW7-16B4, isotype: rat IgG1, κ) antibodies (BioLegend, San Diego, CA, USA), diluted in PermWash at a concentration of 5 mg/mL, were added. Cells were incubated with the antibodies at room temperature for 30 min in the dark and were then washed and fixed with 300 µL of 1% paraformaldehyde. Samples were kept at 4 °C in the dark until flow cytometry analysis, as described below.

### 4.7. Flow Cytometry Analysis

The samples were analyzed with a Cytek Aurora flow cytometer (Cytek Biosciences, Fremont, CA, USA). For γδ T cells, a total of 200,000 events from the lymphocyte gate, selected based on size (FSC) and granularity (SSC), were analyzed. For macrophages, a total of 5000 events from the M1 or M2 gates were analyzed. Data were analyzed with FlowJo version 10.10 (Becton Dickinson, San Jose, CA, USA). Results are reported as absolute cell numbers, determined through Neubauer chamber cell counting and flow cytometry analysis. Unstained cells, compensation controls, compensation beads (UltraComp eBeads, Invitrogen, ThermoFisher Scientific, Waltham, MA, USA), and fluorescence-minus-one controls were used as appropriate.

### 4.8. Statistical Analysis

Statistical analysis was performed with GraphPad Prism 10 (GraphPad Software, Boston, MA, USA). The ELISA results are presented as mean and standard deviation. The anti-NPA antibody titers of mice that received NPA-bearing liposomes or Tris–saline solution were compared with the Mann–Whitney test, with significance set at *p* ≤ 0.05. For flow cytometry, results are presented as mean and standard deviation. Cell numbers of mice that received NPA-bearing liposomes or Tris–saline solution were compared with the Mann–Whitney U test, with significance set at *p* ≤ 0.05.

## 5. Conclusions

In the mouse model of lupus induced by the administration of NPA-bearing liposomes, spleen and mesenteric lymph node γδ T cells produce IFNγ and IL-17 and may play a role in inducing the germinal centers that produce anti-NPA IgG antibodies. In addition, spleen macrophages are polarized towards a proinflammatory M1 phenotype and produce IL-6, a cytokine that amplifies the immune response but can also lead to tissue damage if not adequately regulated. This work contributes to understanding the cellular and molecular mechanisms underlying the production of anti-lipid antibodies and the pathogenesis of SLE.

## Data Availability

The original contributions presented in this study are included in the article and Supplementary Materials. Further inquiries can be directed to the corresponding authors.

## Supplementary Materials

The following supporting information can be downloaded at: https://www.mdpi.com/article/10.3390/ijms26178680/s1.

## Abbreviations

The following abbreviations are used in this manuscript:

γδ T cells	Gamma delta T cells
ARG-1	Arginase 1
IFNγ	Interferon gamma
IL-4	Interleukin 4
IL-6	Interleukin 6
IL-10	Interleukin 10
IL-17	Interleukin 17
MGL	Macrophage galactose-type lectin
MHC	Major histocompatibility complex
MLN	Mesenteric lymph nodes
NPA	Non-bilayer phospholipid arrangements
SLE	Systemic lupus erythematosus
TCR	T cell receptor
TGFβ	Transforming growth factor beta
