# Supplementary material for: In Vivo Response of γδ T Cells and Macrophages to Non-Bilayer Phospholipid Arrangements in a Lupus-like Mouse Model
Source: Int J Mol Sci. 2025 Sep 5;26(17):8680. doi: 10.3390/ijms26178680 (PMC12429272; doi:10.3390/ijms26178680)
Supplement: Supplementary file 1 [file ijms-26-08680-s001.zip › ijms-3820648 Figures data/Figure 1/Fig 1 Nano Liposomes.pdf]

NANOSIGHT

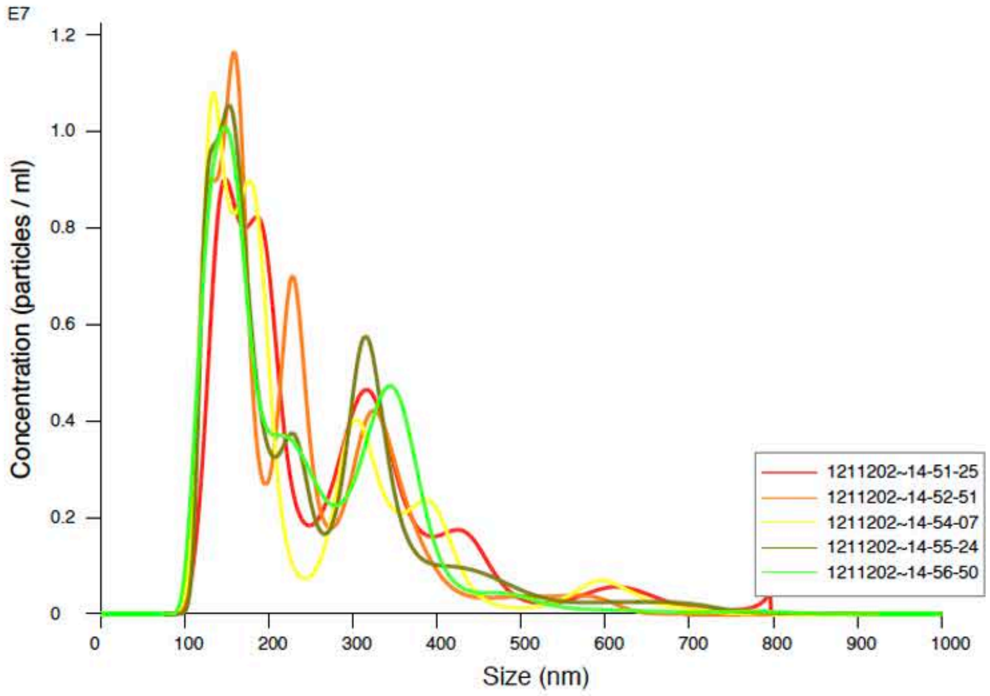

FTLA Concentration / Size graph for Experiment:
12112020 2020-11-12 14-51-08

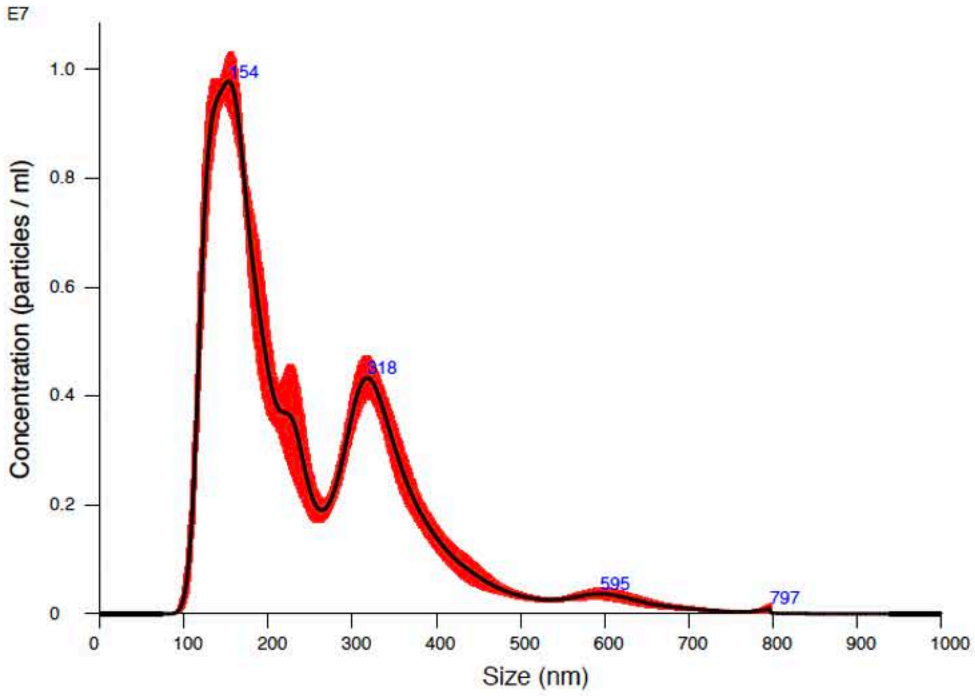

Averaged FTLA Concentration / Size for Experiment:
12112020 2020-11-12 14-51-08
Error bars indicate + / -1 standard error of the mean

Included Files

12112020 2020-11-12 14-51-25
12112020 2020-11-12 14-52-51
12112020 2020-11-12 14-54-07
12112020 2020-11-12 14-55-24
12112020 2020-11-12 14-56-50

Details

NTA Version:	NTA 3.2 Dev Build 3.2.16
Script Used:	SOP Standard Measurement 02-51-08PM 12Nov2020.txt
Time Captured:	14:51:08 12/11/2020
Operator:	PBS
Pre-treatment:	
Sample Name:	
Diluent:	
Remarks:	

Capture Settings

Camera Type:	sCMOS
Laser Type:	Blue488
Camera Level:	11
Slider Shutter:	890
Slider Gain:	146
FPS	25.0
Number of Frames:	1498
Temperature:	21.2 ºC
Viscosity:	(Water) 0.969 - 0.971 cP
Dilution factor:	Dilution not recorded

Analysis Settings

Detect Threshold:	5
Blur Size:	Auto
Max Jump Distance:	Auto: 8.9 - 9.6 pix

Results

Stats: Merged Data

Mean:	246.6 nm
Mode:	153.0 nm
SD:	119.2 nm
D10:	131.1 nm
D50:	204.4 nm
D90:	393.1 nm

Stats: Mean +/- Standard Error

Mean:	246.4 +/- 5.4 nm
Mode:	148.5 +/- 4.1 nm
SD:	118.2 +/- 5.2 nm
D10:	131.5 +/- 2.2 nm
D50:	205.6 +/- 4.6 nm
D90:	394.9 +/- 12.4 nm
Concentration (Upgrade):	1.41e+009 +/- 1.61e+007 particles/ml
	170.5 +/- 5.2 particles/frame
	188.0 +/- 6.4 centres/frame

NANOSIGHT

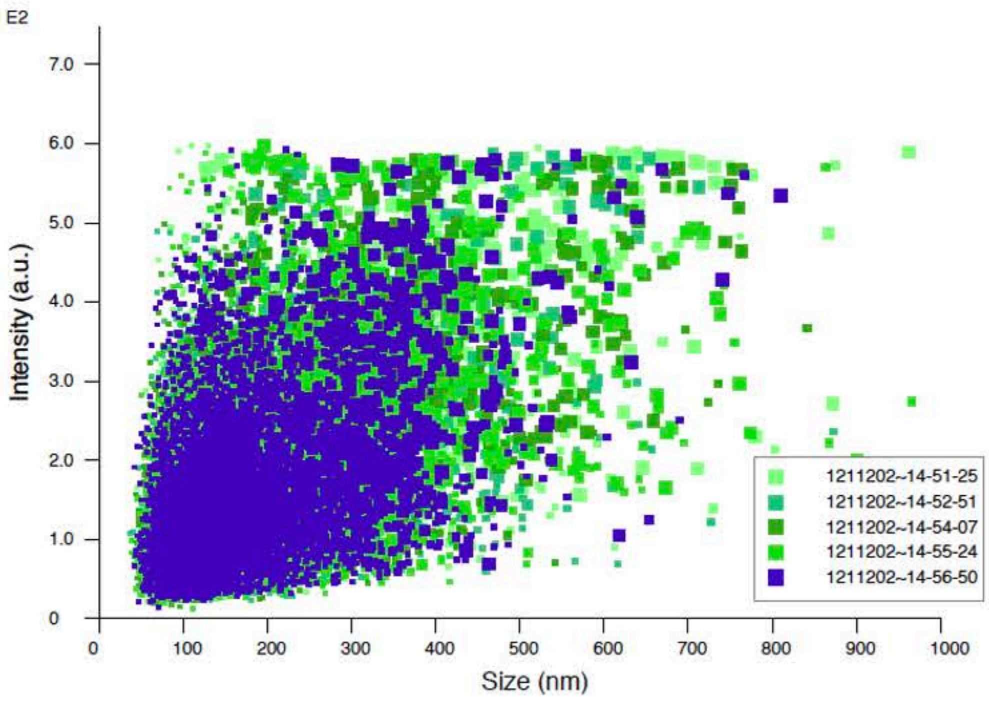

Intensity / Size graph for Experiment:
12112020 2020-11-12 14-51-08
