# Supplementary material for: In Vivo Response of γδ T Cells and Macrophages to Non-Bilayer Phospholipid Arrangements in a Lupus-like Mouse Model
Source: Int J Mol Sci. 2025 Sep 5;26(17):8680. doi: 10.3390/ijms26178680 (PMC12429272; doi:10.3390/ijms26178680)
Supplement: Supplementary file 1 [file ijms-26-08680-s001.zip › ijms-3820648 Figures data/Figure 1/Fig 1 Nano NPA-liposomes.pdf]

NANOSIGHT

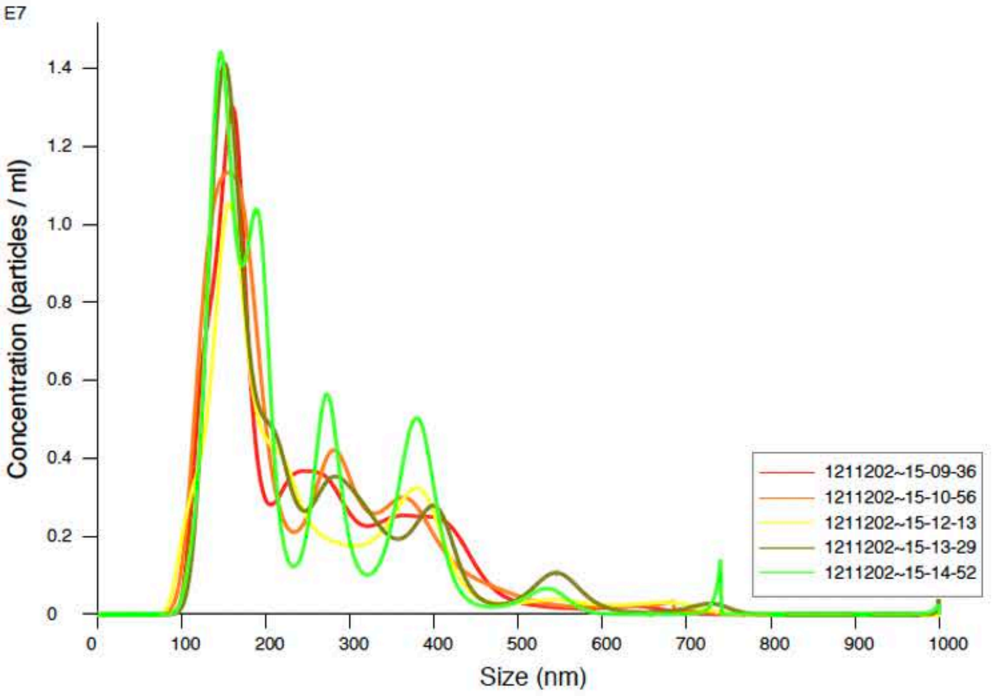

FTLA Concentration / Size graph for Experiment:
12112020 2020-11-12 15-08-55

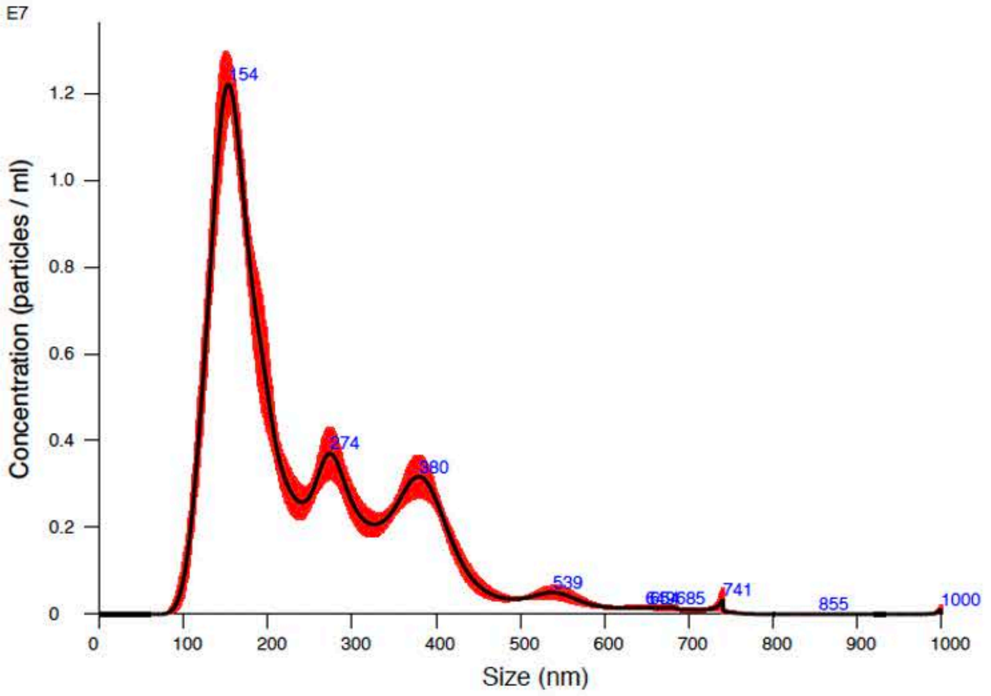

Averaged FTLA Concentration / Size for Experiment:
12112020 2020-11-12 15-08-55
Error bars indicate + / -1 standard error of the mean

Included Files

12112020 2020-11-12 15-09-36
12112020 2020-11-12 15-10-56
12112020 2020-11-12 15-12-13
12112020 2020-11-12 15-13-29
12112020 2020-11-12 15-14-52

Details

NTA Version:	NTA 3.2 Dev Build 3.2.16
Script Used:	SOP Standard Measurement 03-08-55PM 12Nov2020.txt
Time Captured:	15:08:55 12/11/2020
Operator:	2G
Pre-treatment:	
Sample Name:	
Diluent:	
Remarks:	

Capture Settings

Camera Type:	sCMOS
Laser Type:	Blue488
Camera Level:	11
Slider Shutter:	890
Slider Gain:	146
FPS	25.0
Number of Frames:	1498
Temperature:	21.7 - 21.9 °C
Viscosity:	(Water) 0.955 - 0.959 cP
Dilution factor:	Dilution not recorded

Analysis Settings

Detect Threshold:	5
Blur Size:	Auto
Max Jump Distance:	Auto: 9.6 - 10.1 pix

Results

Stats: Merged Data

Mean:	244.6 nm
Mode:	153.1 nm
SD:	118.9 nm
D10:	133.6 nm
D50:	196.5 nm
D90:	401.3 nm

Stats: Mean +/- Standard Error

Mean:	244.8 +/- 2.2 nm
Mode:	154.5 +/- 2.4 nm
SD:	118.9 +/- 2.9 nm
D10:	133.3 +/- 1.4 nm
D50:	198.0 +/- 2.5 nm
D90:	402.3 +/- 4.4 nm
Concentration (Upgrade):	1.50e+009 +/- 4.90e+007 particles/ml
	189.7 +/- 4.4 particles/frame
	202.9 +/- 3.5 centres/frame

NANOSIGHT

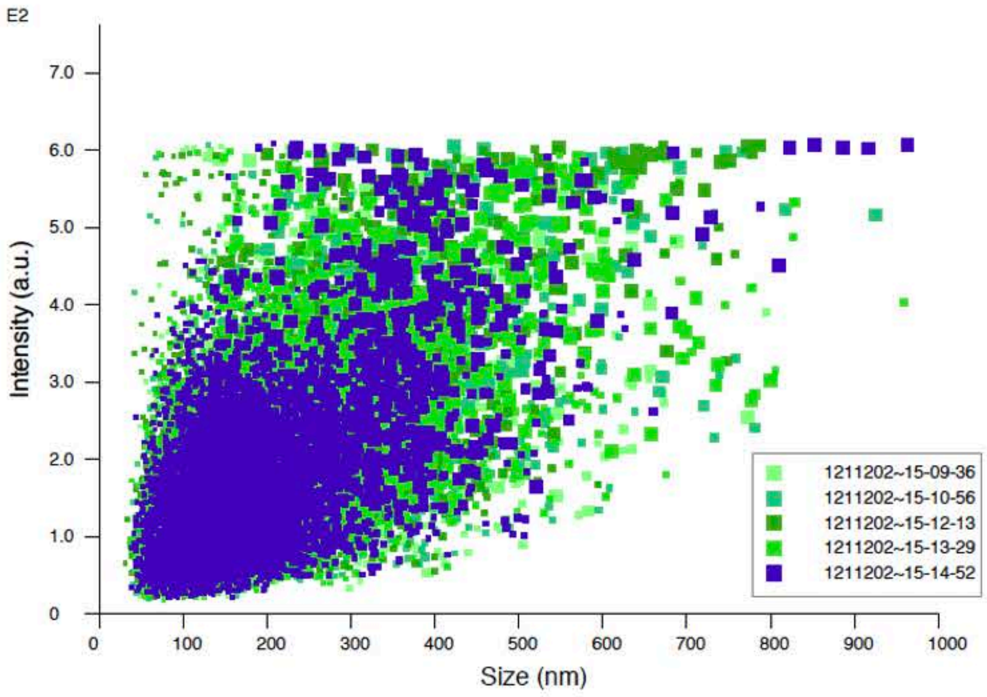

Intensity / Size graph for Experiment:
12112020 2020-11-12 15-08-55
